# Characterization of Alpha-Bungarotoxin Antibodies Prepared by Different Strategies

**DOI:** 10.3390/toxins17120601

**Published:** 2025-12-16

**Authors:** Huijuan Lu, Guowen Zhang, Lin Zhao, Ying Yuan, Bing Gong, Bin Han, Wen-Hui Lee

**Affiliations:** 1 Engineering Laboratory of Peptides of Chinese Academy of Sciences, Key Laboratory of Bioactive Peptides of Yunnan Province and State Key Laboratory of Genetic Evolution & Animal Models, Kunming Institute of Zoology, Chinese Academy of Sciences, Kunming 650201, China; luhuijuanwangyi@163.com (H.L.); zhangguowen@mail.kiz.ac.cn (G.Z.); linzhao7010@163.com (L.Z.); 2 Department of Emergency, The First People’s Hospital of Yunnan Province, The Affiliated Hospital of Kunming University of Science and Technology, Kunming 650034, China; km_ostrich@163.com (Y.Y.); bing_xiahai@163.com (B.G.)

**Keywords:** alpha-bungarotoxin, antibody titer, antibody preparation, snake venom three-finger toxin

## Abstract

The preparation of an antibody to treat snake envenomation requires a large amount of snake venom. In China, only four types of anti-snake venom sera are clinically available, and the production and immunization strategies for clinically approved anti-snake venom sera still mainly rely on detoxified antigens, which is a mature technical route commonly adopted by domestic pharmaceutical enterprises. At present, researchers immunize animals with low doses of certain snake venom toxic components or prokaryotically expressed toxic components to reduce the amount of venom needed, and use prepared antisera for their specific investigation purposes. However, it is unclear if low-dose immunized antibody titers and toxin-neutralizing activities are consistent with those of high-dose detoxified crude venom immunized antibodies. In this study, we developed a method for the preparation of highly effective rabbit polyclonal antisera while saving a large amount of toxin. Rabbit polyclonal antisera prepared by low-dose natural α-bungarotoxin (α-BGT) had strong neutralizing effects on the toxin itself and achieved the same antibody titers as antisera prepared with high doses of detoxified α-BGT. Antigen of A maltose binding protein (MBP) fused with α-BGT (MBP-α-BGT) expressed in prokaryotes had low antibody titer and low neutralizing activity. This study provides an effective dosage selection guide and methods for the preparation of polyclonal antibodies and antiserum for investigation purposes.

## 1. Introduction

*B. multicinctus* is one of the 10 most venomous snakes in the world and the most venomous snake in Asia, but it mainly distributes in mainland China and neighboring countries [1]. *B. multicinctus* venom is neurotoxic, acting on neuromuscular junctions, and can lead to motor nerve disorders, eyelid droop, respiratory paralysis, or even respiratory failure and death without timely treatment [1,2,3]. The lethal components of the venom are neurotoxic proteins and peptides, including α-bungarotoxin (α-BGT), β-BGT, κ-BGT, and γ-BGT [4]. These bungarotoxins are divided into presynaptic and postsynaptic neurotoxins according to their acting targets. Postsynaptic neurotoxins, including α-, γ-, and κ-neurotoxins, competitively bind to acetylcholine receptors at neuromuscular junctions, blocking the transmission of neurotransmitters [5]. Postsynaptic neurotoxins belong to the snake venom three-finger toxin family and can be further divided into short-chain neurotoxins (containing 60–62 amino acid residues and four pairs of disulfide bonds) and long-chain neurotoxins (with 70–74 amino acid residues and five pairs of disulfide bonds) [6]. Notably, the molecular size of these molecules generally makes them have weak immunogenicity. β-BGT is a presynaptic neurotoxin and the most lethal toxin contained in the venom [6].

Alpha-BGTs are alkaline polypeptides containing mostly basic amino acids and 10 cysteine residues that are involved in the formation of five disulfide bonds. This long-chain postsynaptic neurotoxin consists of 74 amino acids with a three-finger-like structure and a relative molecular weight of around 7500 Da [7]. The three-finger structure of the neurotoxin is generally composed of 60–80 amino acid residues that form a polypeptide chain. The composition and relative position of the amino acid residues have similarity among different snake venom three-finger neurotoxins, and both long- and short-chain postsynaptic neurotoxins possess similar three-dimensional structure [8].

The reported venom yield of *B*. *multicinctus* per bite is approximately 4.6 mg dry weight [1], which is extremely low compared with that of *N. naja atra* (approximately 79 mg dry weight [9]) and *Ophiophagus hannah* (56–153 mg dry weight [10]). The lethal dosage of *B. multicinctus* venom on humans is approximately 1 mg, and the venom injected per bite is far more than the lethal dose [11]. During the COVID-19 situation, the enhanced protection for wild animals by the Chinese government has made it more difficult to obtain enough crude venom or purified specific high-dose antigens for immunizing animals in the routine preparation of antibodies. Given the extremely low venom yield of Bungarus multicinctus (only ~4.6 mg dry weight per bite), the high-dose antigen requirement for traditional antiserum preparation has become a bottleneck. Thus, exploring low-dose antigen immunization strategies to obtain high-potency specific antiserum is of great practical significance for the study and application of its key toxic components (e.g., α-BGT). Furthermore, multiple immunizations require the use of high-dose samples [12,13].

The amount of venom injected after *B. multicinctus* envenomation is much less than that of other venomous snakes. Therefore, the quantity of crude venoms needed in the antivenom production process is particularly vital. Previous studies have shown that the use of low-dose antigens to prepare antibodies has certain effectiveness [14]. To investigate the antigenicity, immunogenicity, and toxin-neutralizing activity of specific snake venom components, researchers need to prepare specific antiserum. For basic research on toxin identification and sequence analysis, mass spectrometry techniques have become the mainstream method. However, it is unclear if the titers and neutralization activities of antibodies obtained by low-dose natural antigen immunization are significantly different from those obtained by traditional immunization. In this study, three kinds of polyclonal antibodies were obtained by immunizing rabbits with α-BGT antigens prepared by different strategies. We compared the titers of the antibodies prepared using the three methods by ELISA and the neutralization activity of the antibodies, by calculating the ED_50_ values in animal protection experiments, to find the most economical method for preparing polyclonal antibodies against snake venom three-finger toxins.

## 2. Results

### 2.1. Acquisition of MBP-α-BGT

**Figure 1:**
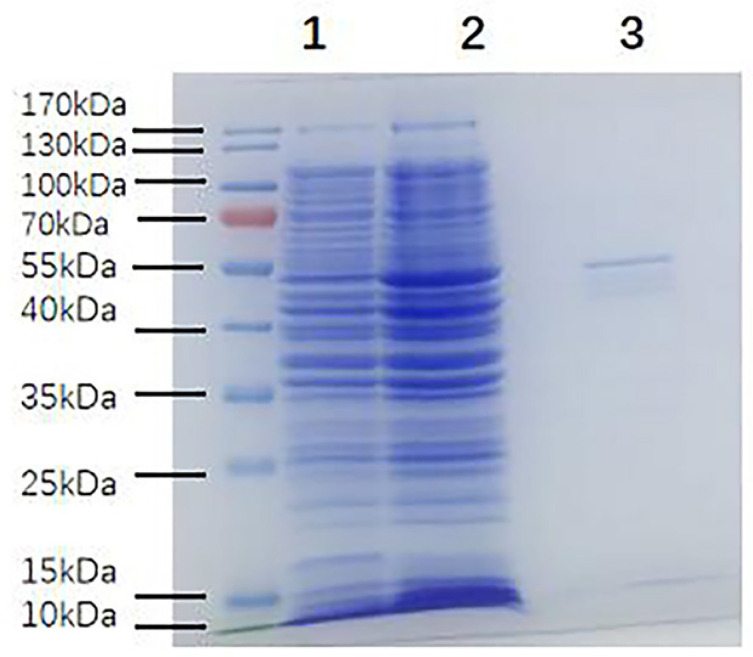
SDS-PAGE of MBP-α-BGT fusion protein expression and purification. SDS-PAGE of bacterial proteins before and after induction and extraction, and under reducing conditions. There were obvious target bands of 55 kDa in the induced extract. 1: Before microbial induction; 2: After microbial induction; 3: After induction and extraction.

To determine whether MBP-α-BGT was induced or not, we compared the bacteria-expressed proteins before and after induction and extraction by SDS-PAGE. The results showed that a clear 55 kDa band protein was expressed after induction (Figure 1). The 55 kDa band protein was identified as MBP-α-BGT by Western blot.

### 2.2. Specificity of Antisera Prepared by Three Methods

To investigate whether specific antibodies were produced after the third immunization, we collected a small amount of venous blood from the ears of three rabbits and used it as the primary antibody in Western blotting to detect the specificity of antibodies.

**Figure 2:**
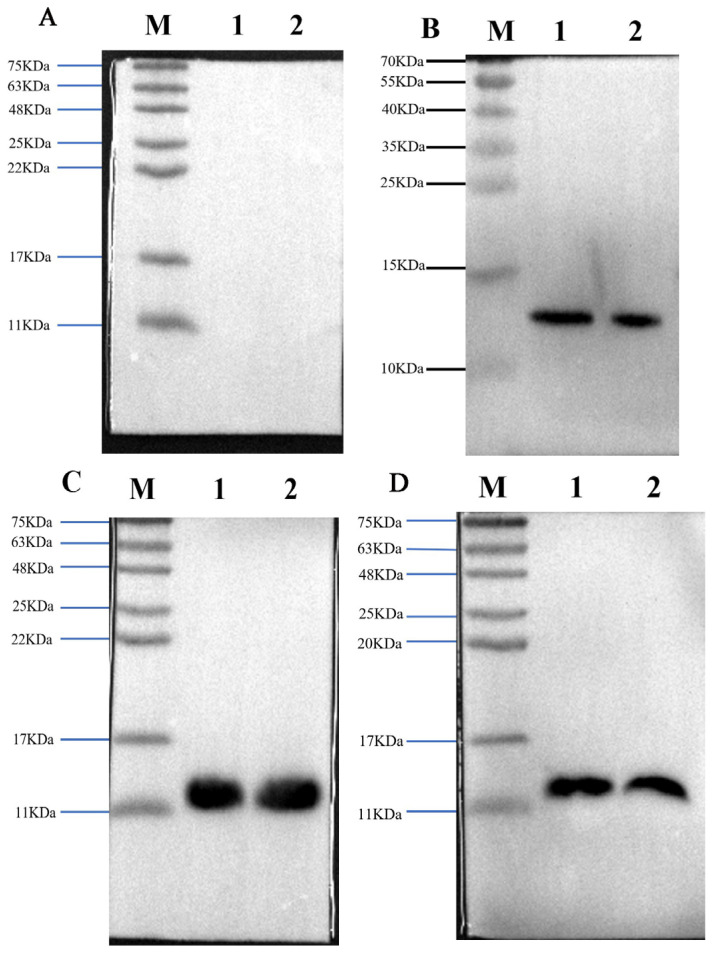
The three laboratory-made antisera recognized antigens and crude toxins with specificity. Western blot profile of preimmunization rabbit serum used as the negative control (**A**); Western blot profile of prepared high-dose α-BGT-immunized antiserum (**B**); Western blot profile of prepared low-dose α-BGT-immunized antiserum (**C**); Western blot profile of prepared MBP-α-BGT antiserum (**D**). In the Western blot, 10 µg of crude venom or 2 µg of purified α-BGT were added to two lanes. 1: α-BGT, 2: *B. multicinctus* crude venom, M: marker.

The results showed that the antisera obtained by three immunization methods specifically recognized *B. multicinctus* crude venom and α-BGT, as strong bands were seen on the Western blot (Figure 2).

### 2.3. Changes in the Three Antisera Titers

To compare changes in the antisera titers, the absorbance values of rabbit sera before immunization and after the third and fourth immunizations were determined by indirect ELISA, measuring the absorbance at 450 nm.

**Figure 3:**
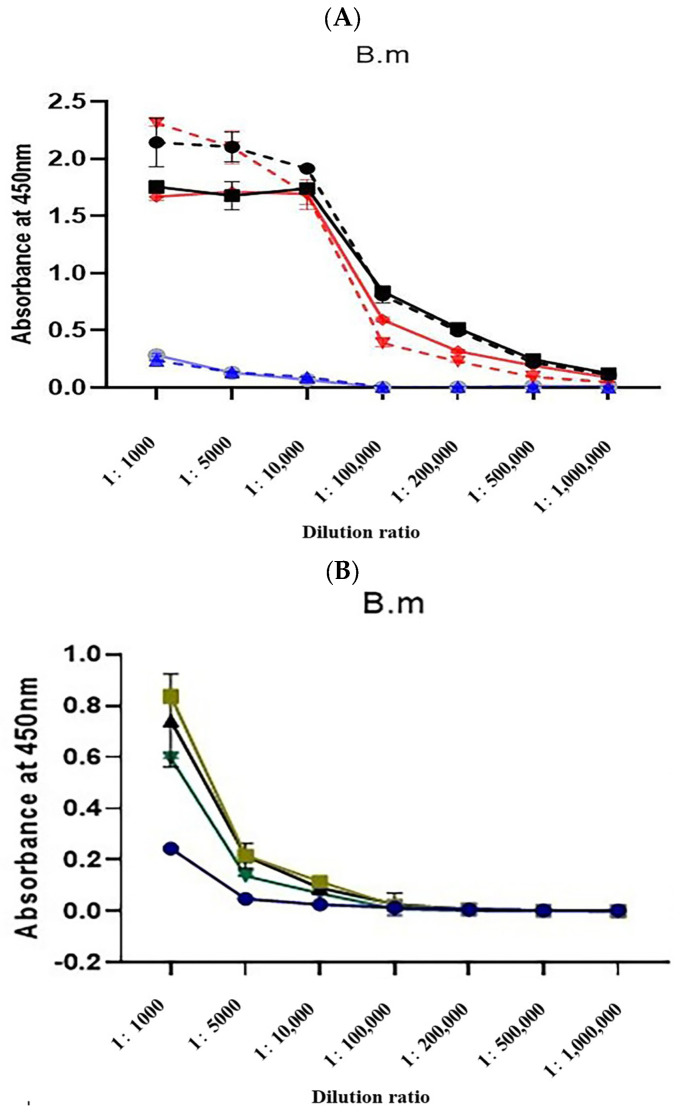
Determination of Antisera Titer (**A**). Absorbance values of rabbit sera collected after the third (dotted line) and fourth immunizations were measured via indirect ELISA. ●: α-BGT antisera after the third immunization. ■: α-BGT antisera after the fourth immunization. ▼: Natural α-BGT antisera after the third immunization. ◆: Natural α-BGT antisera after the fourth immunization. ▲: MBP-α-BGT antisera after the third immunization. ○: MBP-α-BGT antisera after the fourth immunization. The titer of MBP-α-BGT antisera after the fourth, sixth, seventh, and eighth immunization was determined by indirect ELISA (**B**), ●: The fourth MBP-α-BGT antisera. ■: The sixth MBP-α-BGT antisera. ▲: The seventh MBP-α-BGT antisera. ▼: The eighth MBP-α-BGT antisera. Measurement of absorbance at 450 nm. Results are expressed as mean ± SD.

The titers of natural α-BGT antiserum and detoxified α-BGT antiserum reached over 1/500,000 after the third and fourth immunizations, while the titer of the MBP-α-BGT antiserum was only 1/5000 after the third and fourth immunizations (Figure 3A). Further immunization was carried out after boiling and denaturing of the MBP-α-BGT antigen. The sixth and subsequent antiserum titers only increased to 1/10,000 (Figure 3B), and the optimized antiserum titer did not increase significantly.

### 2.4. Antibody Titer Determination

**Figure 4:**
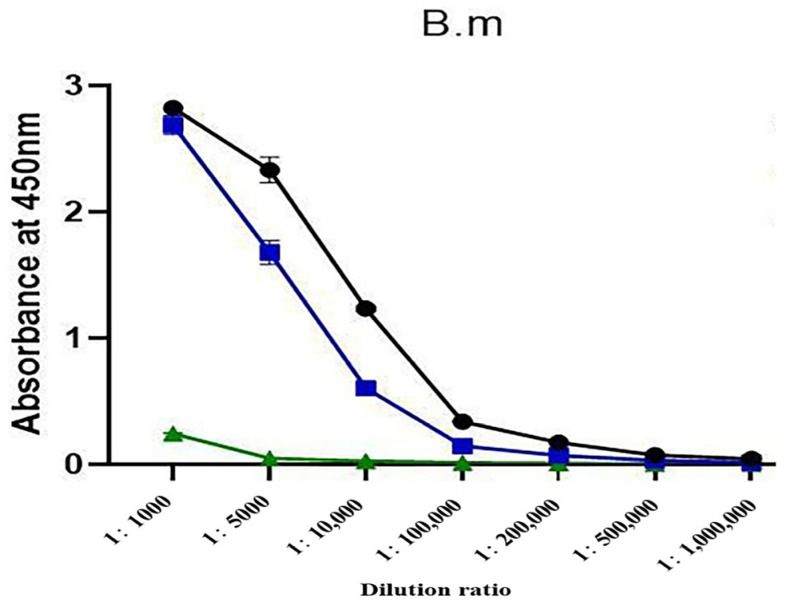
Titer plots of antibodies prepared using three methods were determined by indirect ELISA. ●: High-dose detoxified α-BGT antibody. ■: Low-dose natural α-BGT antibody. ▲: MBP-α-BGT antibody. Measurement of absorbance at 450 nm. Results are expressed as mean ± SD.

Using indirect ELISA, the titers of antibodies prepared using high-dose detoxified antigens and low-dose natural antigens showed no obvious difference (both were 200,000). Under the same conditions, the MBP-α-BGT antibody titer was only 5000 (Figure 4).

### 2.5. Total Antigen Dose for Each of the Three Immunization Methods

The total antigen doses required for α-BGT immunization using the three methods are listed in Table 1.

The low-dose natural α-BGT antibody only required 0.6 mg antigen and four immunization sessions, compared with the 4.8 mg of detoxified the same antigen needed to produce an equal antibody titer. The titer of the low-dose natural α-BGT antibody was the same as that for the high-dose antibody, showing that antigens can be conserved. Immunization with antigens derived from prokaryotic expression produced antibodies with a low titer; however, the advantage of this method is that it does not require natural snake venom (Table 1).

### 2.6. Comparison of Neutralization Activity of the Three Antibodies

To compare the ability of the three antibodies to neutralize the α-BGT toxin, the ED_50_ of the three antibodies and α-BGT was calculated in an animal protection experiment.

This experiment showed that the ED_50_ value of the low-dose natural α-BGT antibody was 11.136, which was close to that of the high-dose detoxified α-BGT antibody (11.14), indicating that the two types of antibodies had similar toxin neutralization and antibody activities. In contrast, the MBP-α-BGT antibody could not neutralize α-BGT and could not protect the mice at 4 mg of antibody per mouse (Table 2).

## 3. Discussion

Snake bite poisoning is a neglected tropical disease resulting in approximately 100,000 deaths a year [15]. The World Health Organization stresses that antivenoms are the only effective antidotes for snake envenomation [16]. In fact, even though antivenoms cure snake envenomation, they are still scarce in many countries [17], especially in poorer areas, where they are prohibitively expensive for some people. The World Health Organization has developed international initiatives to reduce the impact of this neglected tropical disease, one of which is to safeguard global antivenom supplies [18]. At present, the preparation of antibody or antiserum still needs to be optimized because they often lead to allergic reactions and serum disease [19].

Clinically used antivenoms are mostly prepared from serum obtained after immunizing horses with snake venom; a few manufacturers use sheep or donkeys in their production [20,21]. To improve production rates and reduce costs, the most common strategy is to immunize large animals to obtain a large amount of plasma. However, the quantity of snake venom needed to ensure the effective antibody arising in the serum is high. Thus, the crude venom needed is very important [22]. *B. multicinctus* is a highly venomous snake mainly distributed in China, and its injection volume per bite is very small compared with that of other snakes. The reported average dry weight per bite was 4.6 mg when milking *B. multicinctus* venom [1]. Thus, to keep the same antivenom yield as other species (such as cobras), more *B. multicinctus* individuals are needed to obtain enough crude venom, which makes the production of anti-*B. multicinctus* antivenin face more difficulties.

The antibody protection experiment in mice used in this study showed that the neutralization activity of the antibody obtained by immunization with a low dose of natural antigen was consistent with that of the antibody obtained with a high dose of the same detoxified antigen, whereas the MBP-α-BGT antibody did not have the ability to neutralize toxin (Table 2).

In previous studies, broad-spectrum snake antivenom was obtained by immunizing horses with a variety of snake venoms containing non-lethal components and macromolecular toxin components, and the serum neutralized a variety of snake venoms relatively effectively in mouse protection experiments [14]. In 2024, the production of snake antivenom by targeting epidermal dendritic cells via the ‘low-dose, low-volume, multi-site’ immunization strategy was reported by Ratanabanangkoon. He developed a protocol by intradermal injections (2–3 mm depth) of small venom dose (total 1–2 mg venom/400 kg horse) of venom emulsified in CFA (a water-in-oil adjuvant containing mineral oil with the emulsifying agent mannide monooleate and dried heat-killed *Mycobacterium tuberculosis* or *M. butyricum*), into about 20 sites around the neck area of the horse. The effectiveness of this immunization protocol was largely attributed to the bacteria contained in CFA, which target dendritic cells and play a pivotal role in the immune response process. Our previous investigation demonstrated that the clinics used equine antivenin against *B. multicinctus* showed lower recognition ability to crude venom since the antivenin reacted strongly with crude venom under reducing conditions but not under non-reducing conditions. We also reported that our prepared rabbit anti-α-BGT antibodies showed better recognition ability compared with clinically used equine antivenin for both crude venom and purified natural α-BGT [2]. The rabbit anti-a-BGT antibodies were prepared by using a total amount of 4.8 mg detoxified a-BGT as an antigen, as reported previously. To reduce a-BGT consumption and obtain better antibodies to recognize natural antigens, we decided to use a total of 0.6 mg natural a-BGT as an antigen to carry out the present investigations. It should be noted that adding heat-killed *Mycobacterium tuberculosis* or *M. butyricum* in the preparation of toxin emulsion might further reduce the toxic antigen needed in preparing specified antibodies.

At present, only four kinds of equine antivenin are available in China to treat snake bite victims in clinics, and all of them are produced by Shanghai Serum Bio-technology Co., Ltd. We have already demonstrated that Chinese clinically used anti-*B. multicinctus* antivenom mainly recognizes the macromolecular components of the crude venom and neutralizes β-BGT and other macromolecules, but it barely recognizes α-BGT and other low-molecular-weight components. The results of animal protection experiments showed that the ED_50_ values of the *B. multicinctus* antivenom against crude venom, α-BGT, and β-BGT were 17.68 mg/kg, 178.18 mg/kg, and 0.5 mg/kg, respectively, indicating *B. multicinctus* antivenom has few antibody components that can neutralize α-BGT [2]. Alpha-BGT is a neurotoxin that accounts for 10% of *B. multicinctus* crude venom, second only to the neurotoxin content of β-BGT [23]. Certainly, the α-BGT antibody would be beneficial for the treatment of *B. multicinctus* snake bites. In this study, a low dose of α-BGT was used to prepare a high-titer α-BGT antibody, which could provide an optimal method for the preparation of anti-*B. multicinctus* antivenom.

The MBP-α-BGT antibody only weakly recognized crude venom and had a low antibody titer (Figure 4) and low neutralization activity (Table 2). These observations were speculated to be caused by the fact that the antibody obtained after immunization with the MBP tag mainly recognized MBP and relatively weakly recognized α-BGT. Emulsified immunity was carried out after boiling the MBP-α-BGT, and the ELISA results showed that the absorbance increased to some extent at the dilution ratio of 1/1000; this may be due to the high-temperature damage to MBP-α-BGT, which may have resulted in the exposure of more antigen epitope (Figure 3B). From this, we speculated that, during the antibody preparation process, antibodies with high specificity and high titer may be obtained with antigens expressed without labels or with damaged antigen tags.

To quickly prepare antibodies with strong specificity and high titers, a certain amount of antigen epitope exposure is required, and the common method is to immunize animals with a sufficient dose of antigen. Alpha-BGT is highly toxic, and the toxic reaction in rabbits can be reduced by means of formaldehyde detoxification, but the total amount of crude venom required is high. We tried to reduce the amount of antigen required to produce antibodies by immunizing rabbits with the natural α-BGT antigen. The various properties of the prepared low-dose α-BGT antibodies were found to be similar to those of the traditional α-BGT antibodies. We speculated that, because no detoxified treatment is used for small antigen doses, the animals’ immunity is in the natural state, causing the antigen epitope to be more fully exposed and, thus, generating a strong immune response, which makes the antibody easier to capture. This study provides a guide to the selection of methods for optimum antibody preparation. However, the antibody preparation method using low-dose toxic components in this study may be applicable to even lower doses of toxic antigens, as the total 0.6 mg natural α-BGT used was based on our previous experiment with 4.8 mg formalin-detoxified α-BGT for specific antibody production.

## 4. Materials and Methods

### 4.1. Materials

*B. multicinctus* crude venom was obtained from a snake farm in Zhejiang, China. Freeze-dried powdered crude venom was stored at −20 °C. α-BGT was prepared according to our previously reported methods from *B. multicinctus* crude venom with a determined M.W of 7984 Da [2]. Briefly, α-BGT was purified by a combination of Superdex G75 gel filtration, Source 15S ion-exchange, and HPLC C_18_ chromatography column steps.

Kunming mice and rabbits from Hunan SJA Laboratory Animal Co. Ltd (Changsha, China). Bovine Serum Albumin (BSA) was a product of Sigma (Kankakee, IL, USA). ProteinIso^®^ Protein A Resin was purchased from TransGen Biotech Co. Ltd (Beijing, China). Immobilon-P^SQ^ transfer membranes (0.22 µm) were purchased from Merck company (Darmstadt, Germany). HRP-Goat Anti-Rabbit IgG (H + L) was purchased from Proteintech company (Wuhan, China). Corning 96 polystyrene microplates, High-sig ECL Western blotting Substrate, and BCA Protein Quantification Kit were purchased from Thermo Fisher company (Waltham, MA, USA).

### 4.2. Methods

#### 4.2.1. Animals and Ethics Statement

Rabbit (2 kg) and Male Kunming mice (6–8 weeks, 20 g ± 2 g) were provided by Hunan SJA Laboratory Animal Co., Ltd. All animals are free to drink, eat, and move around in a clean environment. All experiments on animals meet the requirements of the National Institutes of Health guide for the care and use of Laboratory animals (NIH Publications No. 8023) and have been reviewed and approved by the Animal Protection and Use Committee of Kunming Institute of Zoology, Chinese Academy of Sciences (Approval ID: SMKX-2017023).

#### 4.2.2. Antigen Detoxification

α-BGT was dissolved in PBS (0.01 M, pH 7.4) containing 0.2% formaldehyde to prepare a 2 mg/mL solution, which was then incubated at 37 °C for 5–7 days. Validation of detoxification effect: Kunming mice were intraperitoneally injected with the detoxified antigen at a dose exceeding 3 times the LD_50_ (200 μL per mouse). If all mice survived for 24 h, the detoxification was considered successful. The detoxified antigen was dialyzed four times using a 3 kDa molecular weight cut-off (MWCO) dialysis membrane against PBS (0.01 M, pH 7.4) at 4 °C to remove formaldehyde.

#### 4.2.3. Protein Determination

The protein concentrations were determined by Pierce BCA assay kit (Thermo Scientific, Rockford, IL, USA) according to the protocol provided by the manufacturer, using Bovine Serum Albumin (BSA) as a standard [24].

#### 4.2.4. Expression of Maltose Binding Protein Fusion α-BGT

Rabbit polyclonal antibodies were obtained by using prokaryotically expressed MBP-α-BGT as an antigen to immunize rabbits. The mature peptide sequence of α-BGT was retrieved from UniProt, and the synthetic gene coding for α-BGT was optimized and inserted into the pMAL-p2X vector (using EcoRI) [New England Biolabs (Beijing) Ltd., Beijing, China] by Shanghai Generay Biotech Co., Ltd. (Shanghai, China). Finally, the MBP-α-BGT expression plasmid was subcloned into *Escherichia coli* (TB1), and the TB1 culture was shaken at 180 rpm and 37 °C. IPTG was added to a final concentration of 0.5 mM when the OD_600_ of the culture medium reached 0.6. The cultures were then grown for 24 h in shaking flasks at 180 rpm and 16 °C. The recombinant MBP-α-BGT protein was successfully expressed in the periplasm of TB1, and the periplasmic fractions were extracted according to the protocols provided by the manufacturer. The fusion protein was then purified using amylose affinity resin [New England Biolabs (Beijing) Ltd., Beijing, China].

#### 4.2.5. Lethality and Dose Conversion

The purified α-BGT LD_50_ value in mice of 0.2 µg/g was taken from previously published data [2], and the LD_50_ dose in mice was converted to 0.074 µg/g in rabbits based on a previously published method [25]. This natural α-BGT dose was immunized into rabbits to produce antibodies in a natural, non-detoxified, low-dose manner.

#### 4.2.6. Methods of Immunizing Rabbits with Three Antigens

The α-BGT used in the experiments was isolated from *B. multicinctus* crude venom. The first immune method was the detoxification of α-BGT with 2% formaldehyde at 37 °C for 5–7 days, and each immunization dose was 1 mg. The second immunization method adopted the method of non-detoxified immunization, in which the antigen was in a natural state, and the dose was 148 µg each time. The third method used prokaryotic expressed non-toxic MBP-α-BGT for non-detoxified immunity, and the antigen dose was 1 mg each time. All three antigens were used to immunize rabbits in the same way. For the first immunization, complete Freund’s adjuvant was mixed with the sample at a volume of 1:1 to form an oil-in-water emulsion and injected subcutaneously into multiple sites on the rabbits’ backs. The second immunization was carried out 10 days after the first, and the incomplete adjuvant and antigen were mixed into an oil-in-water emulsion at a ratio of 1:1. After that, immunizations were performed once every 7 days for four times in all three ways, and the antigen injection method was the same as for the second immunization. Normally, 3 to 4 sites were selected for antigen injection. The antisera titers were not enhanced quickly for MBP-α-BGT as an antigen. Thus, purified MBP-α-BGT was used to immunize animals to elicit antibodies according to the above-mentioned procedures.

#### 4.2.7. Western Blotting Assessment of Antisera Specificity

SDS-PAGE was mainly operated according to the method of the article [26]. Briefly, the mixture of crude venom or α-BGT samples were separately prepared by mixing with buffer (6 × SDS Protein Loding Buffer) in a buffer-to-sample ratio of 1:5. The mixture was boiled for 10 min and centrifuged, and the electrophoresis gel was placed in the electrophoresis buffer solution and run on a 15% SDS-PAGE gel. When the protein ladders were completely separated, the electrophoresis was stopped and prepared for the transfer. A PVDF membrane was immersed in methanol for 15–30 s and fixed in place in the order of black transfer clip, sponge, filter paper, electrophoretic gel, 0.22 µm PVDF membrane, filter paper, sponge, and white transfer clip, and placed in the transfer tank. When wet transfer was completed, the membrane was immersed in 5% skim milk, blocked at room temperature. After 2 h, the primary antibody was diluted 1:5000 with TBST (20 mM Tris-HCl, pH 7.4, containing 0.5% Tween-20) and incubated overnight at 4 °C. The membrane was washed three times with TBST. The goat anti-rabbit secondary antibody was diluted 1:5000 and incubated at room temperature for 2 h after being added to the membrane. An appropriate amount of horseradish peroxidase chemiluminescent substrate was added to the membrane, and the results were observed.

#### 4.2.8. Indirect ELISA Determination of Antisera Titers

Indirect ELISA was performed using the methods from a previous study with modifications [27]. Briefly, *B. multicinctus* crude venom was diluted to 10 µg/mL, and 100 mL of the solution was added to each well of the 96-well polystyrene microplates. After incubating at 4 °C overnight, 100 mL 3% BSA was added to each well and maintained at room temperature for 2 h. Then, each well was washed three times with TBST buffer. The initial concentration of the different prepared antibodies was adjusted to 2 mg/mL, and a gradient dilution ranging from 1:1000 to 1:1,000,000 with TBST of the samples was separately added to each well. After incubation at 37 °C for 45 min and three times TBST washing, 100 µL of 1:5000 goat anti-rabbit IgG diluted in TBST was added to each well and incubated at 37 °C for 45 min. Afterward, 100 µL ELISA HRP chromogenic solution (Beyotime, Shanghai, China) was added to each well after TBST washing, and the plates were incubated in the dark for 25–30 min to allow positive color signals to develop. Finally, 50 µL of ELISA termination liquid was added to each well, and the absorbance values at 450 nm were recorded. Each concentration was triplicated.

#### 4.2.9. Purification of Antibodies

After the third immunization, rabbit ear venous blood was collected and centrifuged at 3000 rpm for 30 min, and the supernatant was extracted and used as the primary antibody in Western blotting to verify the presence of specific antibodies. After verifying the corresponding specificity of the antibody, rabbit blood was collected via the heart after the fourth immunization, and the IgG antibody was purified by protein A column after centrifugation to collect the serum. The three antibodies were dialyzed into 0.01 M PBS and concentrated to 2 mg/mL.

#### 4.2.10. Animal Protection Experiment

The LD_50_ of purified α-BGT was verified at 0.2 µg/g. Six mice in each group were prepared, and natural α-BGT at the 3 × LD_50_ value was mixed with the antibodies and supplemented with PBS to 1200 µL. The mixtures were incubated at 37 °C for 30 min before intraperitoneal injection of 200 µL into each mouse. The number of surviving and dead mice at different concentrations of the prepared three α-BGT antibodies within 48 h was recorded. The ED_50_ values were calculated according to the Spearman–Karber equation below [28]:

$${\mathrm{logED}}_{50}={\log X}_{100}-\frac{\mathrm{logFD}}{n}(\sum t-\frac{n}{2})$$

in which logX_100_ = log dose resulting in 100% survival and 100% survival for all higher doses; logFD = the log dilution factor; *n* = number of mice used at each dose level; *t* = number of mice alive at each dose level; S = the sum of mice surviving at every dose level. The average weight of the mice used in the antivenin protection test was 20 g. The ED_50_ values of the antivenoms were expressed as milligrams of antivenom per kilogram of mouse body weight needed to neutralize the challenge dose of venom.

#### 4.2.11. Statistical Analysis

All experiments were performed in triplicate (*n* = 3). Data were presented as “mean ± standard deviation (SD)” and statistically analyzed using GraphPad Prism 10.1 software.

## Figures and Tables

**Table 1:** Total antigen doses were immunized to obtain the three α-BGT antibodies.

Antibodies	Amount of Antigen	Antibody Titers
Detoxifying α-BGT antibody	4.8 mg	250,000
Natural α-BGT antibody	0.6 mg	250,000
MBP-α-BGT antibody	4.8 mg	5000

**Table 2:** ED_50_ values with antibodies prepared in three ways were obtained in mouse protection experiments.

Antiserum	Toxins	LD_50_ (mg/kg) i.p	Antiserum Dose (µg/Mouse)	Number of Mice (*n* = 6)		ED_50_ (mg/kg)	Reference
				Died	Lived		
Detoxifying α-BGT antibody	α-BGT	0.2 µg/g	62.5	6	0	11.14	Lin Bo (2020) [2]
			125	4	2		
			250	4	2		
			500	0	6		
Natural α-BGT antibody			62.5	6	0	11.136	Present work
			125	4	2		
			250	3	3		
			500	1	5		
			1000	0	6		
MBP α-BGT antibody			4000	6	0	>200	Present work
Not added			0	6	0	Not available	Present work

## Data Availability

The original contributions presented in this study are included in the article. Further inquiries can be directed to the corresponding authors.
